# Radiological and Functional Outcomes and Associated Factors After Secondary Intramedullary Nailing Among Patients With Open Fractures of the Lower Limb

**DOI:** 10.7759/cureus.43420

**Published:** 2023-08-13

**Authors:** Maneesh K Maurya, Tarun Solanki, Vivekanand Pal

**Affiliations:** 1 Orthopaedics, KVR Hospital Kashipur, Kashipur, IND

**Keywords:** external fixator, fracture, intramedullary nailing, femur, tibia

## Abstract

Background

Open fractures of the lower limb are serious injuries caused by high-energy trauma that can lead to long-term disability. Initial treatment includes wound debridement, fracture reduction, and external fixation to stabilize bone fragments. Secondary nailing, a surgical technique to provide additional stability, has been shown to promote early mobilization and improve fracture alignment. However, there is a lack of consensus on the optimal timing and technique for secondary nailing. This study aims to evaluate the functional and radiological outcomes of patients who undergo secondary nailing for open fractures of the lower limb.

Methods

The study was a hospital-based prospective study of 53 patients who underwent secondary nailing for open fractures of the lower limbs. Patients aged 18 years or older, with Gustilo-Anderson classification grades 1, 2, or 3 A and B, who underwent wound debridement and external fixator application, followed by conversion to secondary intramedullary nail fixation between January 2019 and December 2021 were included in the study. The primary outcome measures were functional and radiological outcomes at follow-ups, assessed using the Lower Extremity Functional Scale (LEFS) and Radiographic Union Scale for Tibia fractures (RUST) score. Data were collected prospectively and analyzed using Statistical Product and Service Solutions (SPSS) (IBM SPSS Statistics for Windows, Version 25.0, Armonk, NY). Descriptive statistics were used to summarize patient demographics and injury characteristics, and the Student's t-test and analysis of variance (ANOVA) were used to compare continuous variables between groups. The study had a final analysis of 39 patients.

Results

The study reports the baseline characteristics, radiological, and functional outcomes of 39 patients who underwent secondary nailing for open fractures of the lower limb. The majority of the fractures occurred in the tibia (71.8%), with most classified as grade 3 (A and B) (69.2%). At the end of the six-month period after secondary nailing, 74.4% of the fractures had a union. Radiological and functional outcomes showed significant improvement after undergoing secondary nailing. Gender and age group did not have a significant association with the radiological outcome, while the time interval between external fixation and secondary nailing was significantly associated with the radiological outcome at six weeks and three months.

Conclusion

According to a study, secondary nailing is effective in managing lower limb open fractures with good radiological and functional outcomes. The time interval between external fixation and secondary nailing affects radiological outcomes, with longer delays leading to lower RUST scores. Orthopaedic surgeons should consider this factor when planning surgical management. Larger sample sizes and more extended follow-up periods are needed to confirm findings and evaluate the effect of other variables on the outcome.

## Introduction

Open fractures of the lower limb represent a significant and often debilitating injury that can have long-lasting consequences, leading to chronic disability and a diminished quality of life. These fractures typically occur as a result of high-energy trauma such as motor vehicle accidents, falls from considerable heights, or sports-related incidents. The estimated incidence of open fractures of the lower limb is approximately 11 cases per 100,000 individuals per year, with a higher prevalence observed among males and younger age groups [1,2].

The primary objectives in the treatment of open fractures of the lower limb are centered around promoting bone healing, preventing infections, and restoring optimal functionality. The initial management of these fractures typically involves meticulous wound debridement, fracture reduction, and the implementation of external fixation to stabilize the fractured bone fragments and address any associated soft tissue injuries. While external fixation is generally effective in managing open fractures of the lower limb, it may not always provide adequate stability for early mobilization. Additionally, external fixation has been associated with various complications, including pin tract infections, loss of fixation, delayed union, malunion, and non-union, as reported in the existing literature [3].

In recent years, secondary nailing has emerged as a surgical technique aimed at providing additional stability to the fractured bone fragments. This procedure involves the insertion of an intramedullary nail after the initial external fixation, enhancing the mechanical integrity of the fractured limb. Secondary nailing has shown promising results in terms of promoting early mobilization, improving fracture alignment, and reducing the risk of complications such as deep vein thrombosis and pressure sores [4,5]. However, despite its potential benefits, there remains a lack of consensus regarding the optimal timing and technique for performing secondary nailing in cases of open fractures of the lower limb.

To address this issue, several studies have been conducted to evaluate the efficacy of secondary nailing in the management of open fractures of the lower limb. One retrospective study conducted by Chang et al. reported favorable outcomes in patients who underwent early secondary nailing within two weeks of sustaining the injury [6]. Another study by Cho et al. demonstrated that early secondary nailing was associated with a higher rate of fracture union and a shorter hospital stay compared to delayed nailing [7]. These studies have provided valuable insights into the use of secondary nailing; however, there remains a scarcity of data concerning the long-term functional and radiological outcomes of patients who undergo this procedure.

Therefore, the primary aim of this study is to evaluate the functional and radiological outcomes of patients who have undergone secondary nailing for open fractures of the lower limb. By investigating these outcomes, we seek to contribute to the existing body of knowledge and provide further evidence regarding the effectiveness of secondary nailing in the management of open fractures of the lower limb. The findings from this study will help inform clinical decision-making and optimize the treatment strategies for individuals suffering from these complex injuries.

## Materials and methods

Study design

This study was a hospital-based prospective study of patients who underwent secondary nailing for open fractures of the lower limbs. The study was approved by the Institutional Review Board and all patients provided written informed consent.

Patient selection

All patients aged 18 years or older, who had an open fracture of the lower limb (Gustilo-Anderson (GA) classification grades 1, 2, or 3 A and B) and underwent wound debridement and external fixator application, followed by conversion to secondary intramedullary nail fixation between January 2019 and December 2021, were included in the study (n=53). Patients with polytrauma or neurovascular injury or who had missed any scheduled follow-up were excluded from the study.

Sample size calculation

Based on the previous literature, assuming the union rate as 85.7%, with 10% precision, 95% confidence level, a power of 80%, and a significance level of 0.05, the required sample size was calculated to be 50 patients [8].

Surgical technique

**Figure 1:**
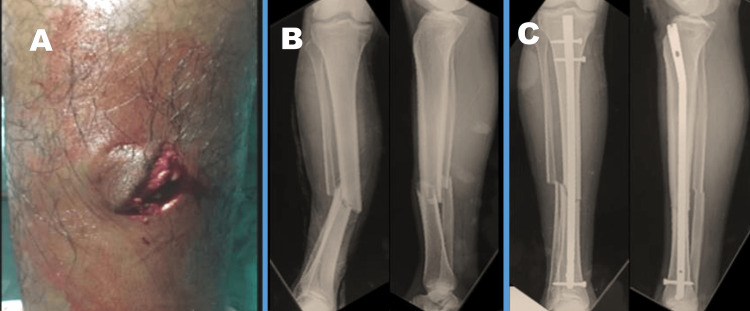
Open fracture of the lower limb. A: Gustilo-Anderson grade 2 fracture. B: Preoperative X-ray of the lower limb. C: Postoperative X-ray of the lower limb.

All surgical procedures were performed by experienced orthopedic surgeons in a standard manner. Under aseptic precautions, thorough debridement of the wound was done and fractures were graded according to GA classification. A two-stage approach was used in all cases, with initial external fixation (monoaxial and biaxial fixation), followed by definitive internal fixation with reamed intramedullary nailing (Figure 1). Once the infection resolved and the condition of the wound, Split Skin Graft (SSG), or flap improved, the external fixators were removed, and the patients were transitioned to either slab or skeletal traction based on the nature of their fracture. Subsequently, once the pin tracts showed granulation, patients were scheduled for definitive fracture fixation. This step-wise approach allowed for adequate healing and preparedness before proceeding with the final stabilization of the fracture and performing the secondary nailing.

Data collection

Data were collected prospectively for all patients. Patient demographics (age, gender) injury characteristics (GA grade, bone involved), surgical details (interval between injury and debridement; interval between external fixation and secondary nailing), and postoperative complications were recorded in a standardized format.

Outcome measures

The primary outcome measures were the functional and radiological outcomes at the follow-ups. The functional outcome was assessed using the Lower Extremity Functional Scale (LEFS) and the radiological outcome was assessed using Radiographic Union Scale for Tibia fractures (RUST) score (determined by the presence of callus and visible fracture line on each of four cortices on anteroposterior and lateral radiographs) [8,9].

Follow-up

**Figure 2:**
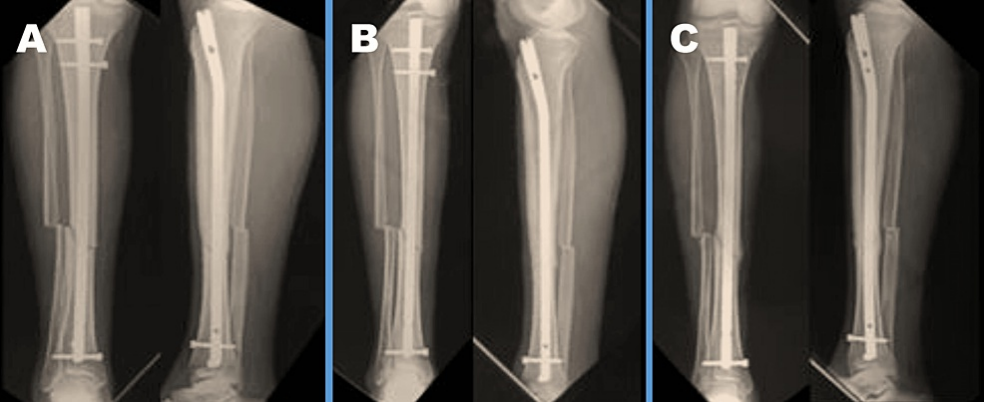
Postoperative X-ray of the lower limb during follow-up at regular intervals. A: Lower limb X-ray at six weeks follow-up period. B: Lower limb X-ray at three months follow-up period. C: Lower limb X-ray at six months follow-up period.

All patients were followed up at regular intervals (six weeks, three months, and six months) after surgery (Figure 2). The follow-up visits included a clinical examination, radiographs, and an assessment of the functional outcome using the LEFS and RUST scores. Any postoperative complications such as infection, nonunion, malunion, or implant failure were noted. During the follow-up period, 14 patients were lost, with seven of them withdrawing from the study and one patient passing away at the five-month mark. Consequently, 39 patients were included in the final analysis of the study.

Data analysis

The collected data were entered into a Microsoft Excel spreadsheet, and statistical analysis was performed using Statistical Product and Service Solutions (SPSS) version 25.0 (IBM Corporation, Armonk, NY, USA). Descriptive statistics were used to summarize the patient demographics and injury characteristics. The continuous variables were presented as mean and standard deviation, while categorical variables were presented as frequencies and percentages. The Student's t-test was used to compare continuous variables between two groups and analysis of variance (ANOVA) was used to continuous variables between three or more groups. A p-value of less than 0.05 was considered statistically significant.

## Results

Table 1 shows the baseline characteristics of the 39 patients who underwent secondary nailing for open fractures of the lower limb. The mean age of the patients was 36.47 ± 12.33 years, with the majority of patients falling within the 30-50 age group (59.0%). There were 33 male patients (84.6%) and six female patients (15.4%). The fractures were primarily located in the tibia (71.8%), with the remaining 28.2% of fractures occurring in the femur. Most of the fractures were classified as grade 3 (A and B) (64.1% and 5.1%), while 23.1% were grade 2 and 7.7% were grade 1. The time interval between injury and debridement varied, with 25.6% of patients receiving debridement within 5 hours of injury, while the remaining 74.4% received debridement after 5 hours. The mean time interval between injury and debridement was 12.65 ± 10.23 hours. The interval between external fixation and secondary nailing also varied, with 17.9% of patients undergoing secondary nailing within 10 days of external fixation and the remaining 82.1% undergoing secondary nailing after 10 days. The mean interval between external fixation and secondary nailing was 34.13 ± 12.68 days. At the end of six months after secondary nailing, only 74.4% (29 patients) of tibia and femur open fractures had a union. The study followed up with the remaining 10 patients beyond the six-month period.

**Table 1:** Baseline characteristics of the patients who underwent secondary nailing for the open fractures of the lower limb (N=39). *ID: time interval between injury and debridement, #ES: interval between external fixation and secondary nailing.

Variables	N/Mean ± SD	%
Mean Age (in years)	36.47 ± 12.33	
Age group		
<30 years	9	23.1
30-50 years	23	59.0
>50 years	7	17.9
Gender		
Male	33	84.6
Female	6	15.4
Fracture of Bone		
Tibia	28	71.8
Femur	11	28.2
Fracture Grade		
1	3	7.7
2	9	23.1
3 A	25	64.1
3 B	2	5.1
Time interval (ID)*		
<5 hours	10	25.6
≥5 hours	29	74.4
Mean Time interval ID (hours)	12.65 ± 10.23	
Time interval (ES)#		
<10 days	7	17.9
≥10 days	32	82.1
Mean Time interval ES (days)	34.13 ± 12.68	
Union rate at the end of follow-up period	29	74.4

Table 2 presents the radiological (RUST) and functional (LEFS) outcome scores of the 39 patients who underwent secondary nailing for open fractures of the lower limb. The RUST score at six weeks was 4.3 ± 1.2, which significantly improved to 5.9 ± 1.4 at three months and further improved to 9.4 ± 2.3 at six months (p-value <0.001). The LEFS score at six weeks was 19.2 ± 7.5, which significantly improved to 33.2 ± 11.6 at three months and further improved to 52.7 ± 13.1 at six months (p-value <0.001). These results suggest that the patients had a significant improvement in both radiological and functional outcomes after undergoing secondary nailing for open fractures of the lower limb.

**Table 2:** Radiological (RUST) and functional (LEFS) outcomes of the patients who underwent secondary nailing for the open fractures of the lower limb (N=39). RUST: Radiographic Union Scale for Tibia fractures, LEFS: Lower Extremity Functional Scale

Score	Mean ± SD	p-value
RUST score (4-12)		
At 6 weeks	4.3 ± 1.2	<0.0001
At 3 months	5.9 ± 1.4	
At 6 months	9.4 ± 2.3	
LEFS (0-80)		
At 6 weeks	19.2 ± 7.5	<0.0001
At 3 months	33.2 ± 11.6	
At 6 months	52.7 ± 13.1	

Table 3 shows the association of different factors with the radiological (RUST) outcome among 39 patients who underwent secondary nailing for open fractures of the lower limb. The results suggest that gender and age group did not have a significant association with the RUST score at any time point. Similarly, fracture type and grade did not show any significant association with the RUST score. However, the time interval between external fixation and secondary nailing was significantly associated with the RUST score at six weeks (p=0.023) and three months (p=0.024). Patients who had a longer time interval (≥10 days) between external fixation and secondary nailing had significantly lower RUST scores at these time points. The time interval between injury and debridement did not show any significant association with the RUST score.

**Table 3:** Association of the factors with the Radiological (RUST) outcome among patients who underwent secondary nailing for the open fractures of the lower limb (N=39). *ID: time interval between injury and debridement, #ES: interval between external fixation and secondary nailing. RUST: Radiographic Union Scale for Tibia fractures

Variables	RUST score (Mean ± SD)			p-value
	At 6 weeks	At 3 months	At 6 months	
Gender				
Male	4.1 ± 0.9	5.7 ± 1.3	9.3 ± 2.1	<0.0001
Female	4.2 ± 1.4	5.9 ± 0.9	9.1 ± 1.8	<0.0001
p-value	0.819	0.721	0.828	-
Age group				
<30 years	4.2 ± 1.5	5.7 ± 1.2	9.2 ± 1.8	<0.0001
30-50 years	4.1 ± .1.1	5.8 ± 0.9	9.3 ± 1.3	<0.0001
>50 years	4.0 ± 0.9	5.8 ± 1.1	9.4 ± 1.1	<0.0001
p-value	0.944	0.967	0.960	-
Fracture of Bone				
Tibia	4.3 ± 0.9	5.8 ± 0.9	9.3 ± 1.7	<0.0001
Femur	4.2 ± 1.1	5.7 ± 1.1	9.2 ± 1.9	<0.0001
p-value	0.771	0.770	0.873	-
Fracture Grade				
1	4.2 ± 1.2	5.8 ± 0.8	9.3 ± 1.2	<0.0001
2	4.2 ± 1.8	5.8 ± 1.7	9.4 ± 1.7	<0.0001
3 (A and B)	4.3 ± 1.5	5.9 ± 1.3	9.4 ± 2.1	<0.0001
p-value	0.984	0.980	0.996	-
Time interval (ID)				
<5 hours	4.3 ± 1.6	5.9 ± 1.2	9.4 ± 2.1	<0.0001
≥5 hours	4.2 ± 1.7	5.8 ± 1.8	9.4 ± 0.7	<0.0001
p-value	0.871	0.872	1.000	-
Time interval (ES)				
<10 days	5.1 ± 1.4	6.3 ± 1.2	9.6 ± 1.8	<0.0001
≥10 days	3.8 ± 1.3	5.2 ± 1.1	9.2 ± 1.5	<0.0001
p-value	0.023	0.024	0.540	-

Table 4 shows the association between different variables and the functional outcome measured by the LEFS among patients who underwent secondary nailing for open fractures of the lower limb. The results show no statistically significant differences in LEFS scores based on gender, age group, fracture site, or fracture grade. However, a significant difference (p=0.044) was found in LEFS scores based on the time interval between external fixation and secondary nailing, with patients who had a shorter time interval (<10 days) showing higher scores at all follow-up periods (six weeks, three months, and six months). There was no significant difference in LEFS scores based on the time interval between injury and debridement.

**Table 4:** Association of the factors with the functional (LEFS) outcome among patients who underwent secondary nailing for the open fractures of the lower limb (N=39). *ID: time interval between injury and debridement, #ES: interval between external fixation and secondary nailing. LEFS: Lower Extremity Functional Scale

Variables	LEFS (Mean ± SD)			p-value
	At 6 weeks	At 3 months	At 6 months	
Gender				
Male	21.4 ± 6.3	35.7 ± 12.2	48.5 ± 12.1	<0.0001
Female	19.1 ± 7.6	39.9 ± 8.9	53.4 ± 14.5	<0.0001
p-value	0.429	0.424	0.381	-
Age group				
<30 years	17.6 ± 6.3	28.1 ± 12.3	55.6 ± 10.1	<0.0001
30-50 years	18.3 ± 6.9	34.3 ± 9.7	52.6 ± 12.3	<0.0001
>50 years	19.6 ± 7.1	31.3 ± 14.1	50.2 ± 12.2	<0.0001
p-value	0.842	0.365	0.658	-
Fracture of Bone				
Tibia	21.3 ± 6.7	33.9 ± 11.2	53.1 ± 12.8	<0.0001
Femur	19.0 ± 8.1	30.1 ± 13.1	51.9 ± 11.3	<0.0001
p-value	0.368	0.369	0.787	-
Fracture Grade				
1	18.9 ± 6.4	36.3 ± 8.9	53.1 ± 14.5	<0.0001
2	21.2 ± 7.6	33.1 ± 11.7	50.3 ± 8.7	<0.0001
3 (A and B)	19.4 ± 5.4	34.2 ± 10.6	47.8 ± 12.2	<0.0001
p-value	0.728	0.903	0.696	-
Time interval (ID)				
<5 hours	20.1 ± 6.4	36.9 ± 5.6	52.4 ± 12.4	<0.0001
≥5 hours	19.1 ± 8.1	32.1 ± 8.9	51.0 ± 8.6	<0.0001
p-value	0.726	0.119	0.695	-
Time interval (ES)				
<10 days	23.2 ± 8.9	36.5 ± 6.9	56.3 ± 6.1	<0.0001
≥10 days	20.4 ± 6.1	32.3 ± 11.8	49.3 ± 8.4	<0.0001
p-value	0.318	0.372	0.044	-

Table 5 presents the occurrence and percentage of complications observed in patients who underwent secondary nailing for open fractures of the lower limb. The reported complications include anterior knee pain (5.1%), pin tract infections (20.5%), pain and discomfort (10.3%), restricted ankle movement (5.1%), pin loosening (5.1%), delayed union (15.4%), and non-union (7.7%).

**Table 5:** Complications outcome among patients who underwent secondary nailing for the open fractures of the lower limb (N=39). *Multiple responses

Complications	Number	%
Anterior knee pain	2	5.1
Pin tract Infection	8	20.5
Pain and discomfort	4	10.3
Restricted ankle movement	2	5.1
Pin loosening	2	5.1
Delayed union	7	17.9
Non-union	3	7.7

## Discussion

Open fractures of the lower limb are among the most common orthopedic emergencies, and their management is often challenging due to the high risk of complications and poor outcomes [10]. In recent years, secondary nailing has emerged as an effective treatment option for these fractures, as it allows for early weight-bearing and restores the mechanical stability of the limb [11]. In this hospital-based prospective study, we evaluated the functional and radiological outcomes of secondary nailing in patients with open fractures of the lower limb.

Our results showed that most of the patients undergoing secondary nailing were males, with a mean age of 36.47 years. The baseline characteristics of the patients in this study revealed that most of the patients were males within the age group of 30-50 years, which is consistent with the findings of previous studies by Court-Brown et al., Anand et al., and Keating et al. [1,12,13]. The majority of fractures were located in the tibia, and most were classified as grade 3 (A and B). These findings are consistent with previous studies that have reported a higher prevalence of tibial fractures [1]. The time interval between injury and debridement varied, with most patients receiving debridement after 5 hours. Similarly, the interval between external fixation and secondary nailing varied, with most patients undergoing secondary nailing after 10 days.

The radiological (RUST) and functional (LEFS) outcome scores of the patients who underwent secondary nailing for open fractures of the lower limb significantly improved from six weeks to three months and six months after surgery. These results are consistent with previous studies by MacKenzie et al., Hertel et al., Gustilo et al., and Pape et al., which have reported good outcomes with secondary nailing in open fractures of the lower limb [11,14-16].

Gender and age group did not show any significant association with the RUST score at any time point. Similarly, fracture type and grade did not show any significant association with the RUST score. This finding is consistent with previous studies by Whelan et al. and Yokoyama et al., which have reported no significant association between these factors and the outcome of secondary nailing in open fractures of the lower limb [17,18]. However, the time interval between external fixation and secondary nailing was significantly associated with the RUST score at six weeks and three months. Patients who had a longer time interval (≥10 days) between external fixation and secondary nailing had significantly lower RUST scores at these time points. This finding is consistent with previous studies by Wu et al. and Blachut et al., that have reported delayed union or nonunion in cases where the time interval between external fixation and secondary nailing is prolonged. The delay in surgery may lead to a prolonged inflammation phase, which could interfere with the healing process [19,20].

In our study, there was no significant difference in RUST and LEFS scores based on the time interval between injury and debridement. Similarly, studies by Li et al., Singh et al., and Reuss et al. have concluded that there is no significant correlation between the timing of initial debridement and the final functional and radiological outcome [21-23].

At the end of six months after secondary nailing, only 74.4% (29 patients) of tibia and femur open fractures had a union, while Wu et al., Blachut et al., and Malik et al. achieved union rates of 95%, 96%, and 94%, respectively, but it is important to note that they achieved these high rates at the end of one year, whereas our study followed the index patients for only six months [19,20,24].

In a study conducted by Steinber et al., involving 54 cases with diaphyseal fractures, it was found that 20.4% of patients experienced complications [25]. However, in our study, a higher percentage of patients (28.2%) developed complications. Specifically, delayed union was observed in 17.9% of patients in our study, whereas Kumar et al., reported a lower incidence of 5.4% [26]. Additionally, non-union occurred in 7.7% of patients in our study, while Kumar et al., reported a slightly higher rate of 8.1% [26]. Bonnevialle et al. conducted a study on intramedullary nailing with reaming (Grosse-kempf nail) involving 32 patients, in which only one case (3.12%) developed a deep infection. In contrast, our study revealed a higher rate of infections, with 20.5% of patients experiencing infection [27]. Furthermore, Steinber et al. reported that 9.3% of patients in their study developed an infection, with 5.55% experiencing deep infections and 3.7% developing superficial infections [25]. These findings highlight the variation in complication rates among different studies, emphasizing the importance of further research and evaluation to improve the understanding and management of complications associated with intramedullary nailing for open fractures of the lower limb.

The strength of our study is its prospective design, which allowed us to obtain accurate and reliable data. However, our study has some limitations. Firstly, the sample size is small, which limits the generalizability of our findings. Secondly, the study was conducted in a single center, which may limit the external validity of the results. Also, the follow-up period was shorter (i.e. six months), so long-term complications were not captured. In our study, one notable limitation is the absence of a control group, which makes it challenging to compare the outcomes of secondary nailing with alternative treatment approaches. This limitation weakens the study's ability to establish the effectiveness of secondary nailing definitively. Without a control group, it is difficult to ascertain whether the observed complications and outcomes are specifically attributable to secondary nailing or if they could be influenced by other factors. Therefore, further research with a control group is warranted to provide a more comprehensive understanding of the relative effectiveness and potential benefits of secondary nailing in the management of open fractures of the lower limb. Thirdly, we did not evaluate the effect of other variables such as smoking, diabetes, or obesity, which may influence the outcome of secondary nailing in open fractures of the lower limb. Future studies should consider these variables to better understand the factors that influence the outcomes of secondary nailing in patients with open fractures of the lower limb.

## Conclusions

Our study suggests that secondary nailing is an effective technique for managing open fractures of the lower limb, with good radiological and functional outcomes. The time interval between external fixation and secondary nailing is an essential factor that influences the radiological outcome, with longer delays leading to lower RUST scores. Orthopedic surgeons should consider this factor when planning the surgical management of open fractures of the lower limb. Future studies with larger sample sizes and more extended follow-up periods are needed to confirm our findings and evaluate the effect of other variables on the outcome of secondary nailing in open fractures of the lower limb.

## References

[1] Court-Brown CM, Caesar B (2006). Epidemiology of adult fractures: a review. Injury.

[2] Karbakhsh M, Mousavi H, Abedzadeh-Kalahroudi M (2017). Epidemiology of traumatic fractures in Iran: A systematic review. Injury.

[3] Ekeland A, Thoresen BO, Alho A, Strömsöe K, Follerås G, Haukebø A (1988). Interlocking intramedullary nailing in the treatment of tibial fractures: a report of 45 cases. Clin Orthop Relat Res.

[4] Browner BD, Jupiter JB, Levine AM, Trafton PG, Krettek C (2010). Skeletal Trauma Basic Science, Management, and Reconstruction Fourth Edition + DVD. Eur J Orthop Surg Traumatol.

[5] Altay M, Ertürer E, Akman S (2012). Functional outcome of patients with Gustilo type III open tibial fractures treated with primary and secondary nailing. Acta Orthop Traumatol Turc.

[6] Chang SM, Song WK, Kang YG, Kim BS, Kim TY, Kim YH (2013). Early secondary nailing for open tibial fractures: can we predict when the soft tissue condition is ready?. Arch Orthop Trauma Surg.

[7] Cho JW, Kim JH, Kim KI, Song KS (2019). Timing of reamed intramedullary nailing in open tibial fractures: a propensity score-matching analysis. Injury.

[8] Whelan DB, Bhandari M, Stephen D, Kreder H, McKee MD, Zdero R, Schemitsch EH (2010). Development of the radiographic union score for tibial fractures for the assessment of tibial fracture healing after intramedullary fixation. J Trauma.

[9] Binkley JM, Stratford PW, Lott SA, Riddle DL (1999). The Lower Extremity Functional Scale (LEFS): scale development, measurement properties, and clinical application. North American Orthopaedic Rehabilitation Research Network. Phys Ther.

[10] Bhandari M, Guyatt GH, Swiontkowski MF, Tornetta P 3rd, Sprague S, Schemitsch EH (2002). A lack of consensus in the assessment of fracture healing among orthopaedic surgeons. J Orthop Trauma.

[11] MacKenzie EJ, Bosse MJ, Kellam JF (2002). Factors influencing the decision to amputate or reconstruct after high-energy lower extremity trauma. J Trauma.

[12] Anand A, Harjai M, Sud A (2022). Functional and radiological outcome after secondary nailing in open fractures of lower limbs: a hospital based prospective study. J Clin Orthop Trauma.

[13] Keating JF, O'Brien PI, Blachut PA, Meek RN, Broekhuyse HM (1997). Reamed interlocking intramedullary nailing of open fractures of the tibia. Clin Orthop Relat Res.

[14] Hertel R, Pisan M, Lambert S (1993). Closed suction drainage of the knee joint. Is it necessary?. Am J Sports Med.

[15] Gustilo RB, Anderson JT (1976). Prevention of infection in the treatment of one thousand and twenty-five open fractures of long bones: retrospective and prospective analyses. J Bone Joint Surg Am.

[16] Pape HC, Rixen D, Morley J (2007). Impact of the method of initial stabilization for femoral shaft fractures in patients with multiple injuries at risk for complications (borderline patients). Ann Surg.

[17] Whelan DB, Bhandari M, McKee MD (2002). Interobserver and intraobserver variation in the assessment of the healing of tibial fractures after intramedullary fixation. J Bone Joint Surg Br.

[18] Yokoyama K, Uchino M, Nakamura K, Ohtsuka H, Suzuki T, Boku T, Itoman M (2006). Risk factors for deep infection in secondary intramedullary nailing after external fixation for open tibial fractures. Injury.

[19] Wu CC, Shih CH (1993). Complicated open fractures of the distal tibia treated by secondary interlocking nailing. J Trauma.

[20] Blachut PA, Meek RN, O'Brien PJ (1990). External fixation and delayed intramedullary nailing of open fractures of the tibial shaft. A sequential protocol. J Bone Joint Surg Am.

[21] Li J, Wang Q, Lu Y, Feng Q, He X, Li Md Z, Zhang K (2020). Relationship between time to surgical debridement and the incidence of infection in patients with open tibial fractures. Orthop Surg.

[22] Singh A, Jiong Hao JT, Wei DT, Liang CW, Murphy D, Thambiah J, Han CY (2018). Gustilo IIIB open tibial fractures: an analysis of infection and nonunion rates. Indian J Orthop.

[23] Reuss BL, Cole JD (2007). Effect of delayed treatment on open tibial shaft fractures. Am J Orthop (Belle Mead NJ).

[24] Malik MH, Harwood P, Diggle P, Khan SA (2004). Factors affecting rates of infection and nonunion in intramedullary nailing. J Bone Joint Surg Br.

[25] Steinberg EL, Geller DS, Yacoubian SV, Shasha N, Dekel S, Lorich DG (2006). Intramedullary fixation of tibial shaft fractures using an expandable nail: early results of 54 acute tibial shaft fractures. J Orthop Trauma.

[26] Kumar R, Sujai S, Chethan NH, Swamy S (2017). Results of open fractures of tibia treated by external fixator as primary and definitive procedure. Int J Ortho Sci.

[27] Bonnevialle P, Cariven P, Bonnevialle N, Mansat P, Martinel V, Verhaeghe L, Mansat M (2003). Segmental tibia fractures: a critical retrospective analysis of 49 cases [Article in French]. Rev Chir Orthop Reparatrice Appar Mot.

